# Characterization and Pathogenicity of a Porcine Reproductive and Respiratory Syndrome Virus Strain with Strong Homology to a HP-PRRSV Vaccine Strain in the Field

**DOI:** 10.1155/2024/1297975

**Published:** 2024-06-21

**Authors:** Chunhua Wei, Xin Lan, Wenlin Huang, Yuan Yang, Hui Yu, Chen Liu, Cuiqin Huang, Manlin Luo, Jiankui Liu

**Affiliations:** ^1^ College of Life Sciences Longyan University, Longyan 364012, Fujian, China; ^2^ Fujian Provincial Key Laboratory for the Prevention and Control of Animal Infectious Diseases and Biotechnology Longyan University, Longyan 364012, Fujian, China; ^3^ Engineering Research Center for the Prevention and Control of Animal Original Zoonosis Fujian Province University College of Life Science Longyan University, Longyan 364012, Fujian, China; ^4^ College of Animal Science Fujian Agriculture and Forestry University, Fuzhou 350002, Fujian, China; ^5^ College of Veterinary Medicine South China Agricultural University, Guangzhou 510642, Guangdong, China

## Abstract

A PRRSV strain, PRRSV2/CN/FJLX06/2021, with strong homology to an MLV-like strain HeN1201 that evolved from the highly pathogenic PRRSV vaccine virus HuN4-F112, was isolated from a dying piglet in China. BLAST and phylogenetic analyses showed that PRRSV2/CN/FJLX06-2021 was most closely related to HeN1201 and HuN4 (the parental strain of MLV HuN4-F112) and clustered with Chinese HP-PRRSV strains in PRRSV-2 lineage 8.7. Importantly, 29 of the 39 characteristic amino acid mutations in the HuN4-F112 genome were found at the corresponding sites of PRRSV2/CN/FJLX06-2021. Animal studies showed that piglets infected with PRRSV2/CN/FJLX06-2021 had a persistent high fever, higher viremia, presence of interstitial pneumonia, and a higher mortality rate (40%) within 2 weeks than those vaccine-inoculated with HuN4-F112. Taken together, these data suggest that PRRSV2/CN/FJLX06-2021 is an MLV-like strain that has evolved from MLV HuN4-F112 and is highly pathogenic to piglets.

## 1. Introduction

Porcine reproductive and respiratory syndrome virus (PRRSV) is an enveloped, single-stranded, positive-sense RNA virus of the genus *Porartevirus* of the family Arteriviridae of the order Nidovirales [1, 2]. Since it emerged in the United States in 1987 [3] and Europe in 1991 [4], PRRSV has rapidly spread to swine-producing countries and has become a devastating disease of pigs worldwide [4, 5, 6, 7, 8]. PRRSV is classified into two distinct species: *Betaarterivirus suid* 1 (PRRSV-1, European species) and *Betaarterivirus suid* 2 (PRRSV-2, North American species) (https://talk.ictvonline.org/taxonomy) [1]. The wide range of genetic and antigenic variation has made the diagnosis and control of PRRSV strains extremely challenging.

The PRRSV genome, including the 5′ untranslated region (UTR) and a poly-A tail at the 3′ end, is approximately 15 kb in size and contains at least 11 open reading frames (ORFs), including ORF1a, ORF1b, ORF2a, ORF2b, ORF3, ORF4, ORF5a, ORF5, ORF6, ORF7, and Nsp2TF [6, 9]. ORF1a and ORF1b, the two largest ORFs, encode at least 16 nonstructural proteins (Nsps), including Nsp1*α*/1*β*, Nsp2TF, Nsp2N, Nsp2-6, Nsp7*α*/*β*, and Nsp8-12. In addition, ORF2a, ORF2b, ORFs3-7, and ORF5a encode a total of eight viral structural proteins known as GP2a, E, GP3, GP4, GP5, M, N, and GP5a [6, 10].

Currently, PRRSV-2 has been prevalent in Chinese swine herds since it was first reported in 1996 and remains the dominant strain in the region. Chinese PRRSV-2 were divided into four different genetic lineages: NADC30-like/NADC34-like (lineage 1), QYYZ-like (lineage 3), VR2332-like (lineage 5), and JXA1-like/CH1a-like (lineage 8) according to the PRRSV classification system [11, 12, 13, 14, 15]. Impressively, a highly pathogenic PRRSV (HP-PRRSV/JXA1-like/sublineage 8.7), represented by the JXA1 strain, with a characteristic discontinuous deletion of 30 amino acids (aa) in Nsp2, emerged in China in 2006, causing significant economic losses to the Chinese swine industry [14, 16, 17]. To prevent and control PRRS, commercial modified-live virus (MLV) vaccines have been widely used in swine herds. Previous studies have shown that HP PRRSV vaccine-like strains are one of the most prevalent strains in Chinese pig herds [18, 19, 20]. In this study, we have isolated and identified a PRRSV strain PRRSV2/CN/FJLX06/2021 in the field, which has evolved from the MLV HuN4-F112 strain (a live attenuated virus vaccine strain derived from HP-PRRSV HuN4). Subsequently, the whole genome of PRRSV2/CN/FJLX06/2021 was sequenced and its pathogenicity was further analyzed in piglets.

## 2. Materials and Methods

### 2.1. Virus Isolation

In October 2022, several litters of newborn piglets showed clinical signs of fever, coughing, and respiratory distress, and approximately 30% of the piglets died in a pig farm in China. Sera collected from the affected piglets were positive for PRRSV by reverse transcription polymerase chain reaction (RT-PCR). The virus was isolated from the positive samples using MARC-145 cells [21] and was confirmed by real-time RT-PCR in accordance with the supplier's instructions (Beijing Anheal Laboratories Co., Ltd.). In addition, indirect immunofluorescence assays (IFA) were carried out to detect PRRSV by polyclonal antibody for PRRSV N protein (Bioss, Beijing, China) as described previously [22]. The purified virus was then used for whole-genome sequencing and animal experiments.

### 2.2. RT-PCR and Genomic Sequencing

Total RNA was extracted from serum samples and MARC-145 cell culture supernatants using a viral nucleic acid extraction kit (Tiangen Biotech (Beijing) Co., Ltd., China), and RT-PCR were carried out as described previously [14]. The whole viral genomes were amplified using eight pairs of specific overlapped primers described in Zhou et al. [23]. Each PCR product was purified and cloned into the pEASY-Blunt vector (TransGen Biotech, China). Recombinant clones were submitted to Biosune Biotechnology Corporation (Fuzhou, China) for sequencing.

### 2.3. Sequence Data Analysis

Thirty-five representative PRRSV strains (Table 1) were used in this study for comparative sequence analyses using the DNASTAR 7.0 package. Multiple sequence alignments of the PRRSVs from this study strains and reference strains were carried out using ClustalW in the MEGA 7.0 software package. Phylogenetic trees with 1,000 bootstrap replicates were constructed from the nucleotide sequences using the neighbor-joining method with MEGA 7.0 software [14, 24].

Potential recombination events were identified using recombination detection program 4.10 (RDP 4.10) [25]. Seven methods (RDP, BootScan, GENECONV, Chimera, MaxChi, SiScan, and 3Seq) in RDP 4.10 were used to detect recombination events and breakpoints. A recombination event was identified when confirmed at least five of the seven mentioned methods with *p* < 0.01.

### 2.4. Animal Experiment

Fifteen 4-week-old piglets tested negative for PRRSV, PCV2, PRV, and CSFV and were assigned to three different groups. The number of piglets used in each group (five piglets/group) was performed according to Wang et al. [24]. Group 1 (*n* = 5) was inoculated intranasally with 2 mL (2 × 10^5^ TCID50) of fifth-passage PRRSV2/CN/FJLX06/2021, while the piglets in group 2 (*n* = 5) were vaccinated intranasally a double dose of HuN4-F112 (2 × 10^5^ TCID50). Piglets in group 3 (*n* = 5) were inoculated with 2 mL DMEM as a negative control.

The rectal temperature of the infected piglets was monitored daily from 0 to 14 days postinoculation (dpi). Serum samples in each group were collected on days 0, 4, 7, 11, and 14 dpi [21]. PRRSV-specific N protein antibody levels in sera of challenged piglets at different days were analyzed using the PRRSV-Abs ELISA kit using cutoff value (S/P) of 0.4 (IDEXX Laboratories Inc., Westbrook, ME, USA). Meanwhile, the serum viremia of the inoculated piglets was assessed by using an IFA microtitration infectivity assay [24]. Piglets were necropsied if they died in the experiment or were euthanized at 14 dpi. The severity of gross lung lesions was estimated and recorded as previously described [26, 27]. Lung tissue from each pig at necropsy was collected and fixed in 10% neutral buffered formalin for hematoxylin and eosin (H&E) staining for histopathological changes and immunohistochemistry (IHC) with polyclonal antibody for PRRSV N protein (Bioss, Beijing, China) [14, 27]. A scoring system (from 0 to 4) was used to assess the overall level of lung microscopic lesion as previously described [26, 27]. As described previously [27], the number of PRRSV antigen-positive cells in lung sections was estimated by a ranked score of 0–4.

### 2.5. Statistical Analysis

The difference analysis on the virus titers and serological (S/P ratio) values was analyzed by using two-way ANOVA of variance at each time point. One-way ANOVA was used to assess the significant difference in lung gross and microscopic lesion scores. All tests were performed using GraphPad Prism 6.01 software, and results were considered statistically significant at a probability (*p*) value < 0.05.

## 3. Results

### 3.1. Genomic Characterization and Phylogenetic Analysis of PRRSV2/CN/FJLX06-2021

PRRSV was successfully isolated from serum samples using MARC-145 cells and was confirmed by IFA (Figure S1) and designated PRRSV2/CN/FJLX06/2021. The genome of PRRSV2/CN/FJLX06/2021 (GenBank No. OQ357724) was 15,320 nt in length excluding the poly (A) tails. The genome alignment showed that the PRRSV2/CN/FJLX06/2021 strain shared 84.5%, 87.7%, 89.5%, 99.2%, and 60.3% nucleotide identity with NADC30 (lineage 1), QYYZ (lineage 3), VR2332 (sublineage 5.1), JXA1 (sublineage 8.7), and LV (PRRSV-1 representative virus), respectively (Table 2). A BLASTn search was performed, and PRRSV2/CN/FJLX06/2021 appeared to share the highest identity (99.7%) with HeN1201 (GenBank no.MF689000) evolved from the HP-PRRSV vaccine strain HuN4-F112. In addition, PRRSV2/CN/FJLX06/2021 also shows a high identity (99.5%) with the Chinese HP-PRRSV strain HuN4 (Table 2). Each region of the PRRSV2/CN/FJLX06/2021 genome was further compared with representative PRRSV-2 of different lineages (Table 2). Notably, we found that PRRSV2/CN/FJLX06/2021 shared the highest amino acid identities with HeN1201 for all fragments, except for Nsp10, GP5, and N protein (Table 2). Compared to prototype VR2332, PRRSV2/CN/FJLX06/2021 had a 30-amino-acid deletion within Nsp2 (aa 481 and aa 533-561), with the same deletion pattern as Chinese HP-PRRSVs (data not shown).

**Figure 1:**
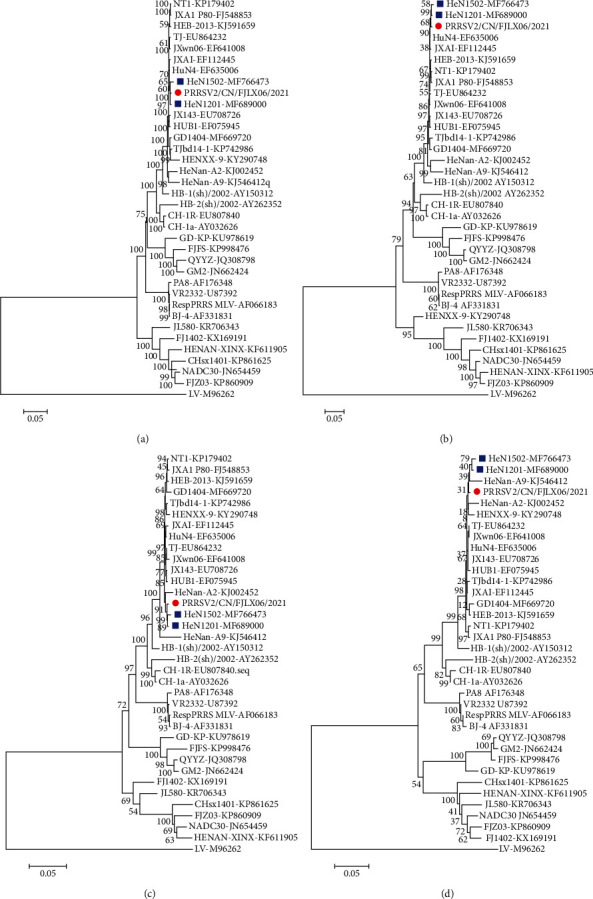
Phylogenetic trees constructed from the nucleotide sequences of the whole genome (a), Nsp2 (b), ORF2-7 (c), and ORF5 (d) of PRRSV2/CN/FJLX06-2021 with the reference PRRSV strains. Isolate PRRSV2/CN/FJLX06-2021 is marked with a red circle in this study. The two representative MLV-like strains evolved from HuN4-F112 are marked with blue rectangles.

To further characterize the genetic evolutionary relationship between PRRSV2/CN/FJLX06/2021 and the 35 representative PRRSVs, phylogenetic analysis was performed based on the whole genome, ORF2-7, Nsp2, and ORF5 nucleotide sequences. The results showed that PRRSV2/CN/FJLX06/2021 was most closely related to HeN1201 and HuN4 and clustered with Chinese HP-PRRSV strains in PRRSV-2 lineage 8.7 (Figure 1). Meanwhile, the recombination analysis showed that no recombination event was observed in the PRRSV2/CN/FJLX06-2021 isolate (Figure S2).

### 3.2. Amino Acid Mutation Analysis of PRRSV2/CN/FJLX06-2021, HeN1201, and HuN4-F112

A previous study showed that MLV HuN4-F112 has 39 unique amino acid mutations distributed in 15 viral proteins compared to HuN4 [28]. On the basis of the highest nucleotide identity of PRRSV2/CN/FJLX06-2021 with the MLV HuN4-F112-like strain HeN1201, the genetic relationship of PRRSV2/CN/FJLX06-2021 with HeN1201 and MLV HuN4-F112 was further analyzed. Comparative analyses of coding region sequences showed that PRRSV2/CN/FJLX06-2021 shared 29 and 33 amino acid mutations identical to HuN4-F112 and HeN1201, respectively (Table 3). Meanwhile, nine reversion mutations (Thr^477^ and Ser^1009^ in Nsp2; Ile^2250^ in Nsp7; Thr^22^ in Nsp9, Lys^702^, and Glu^712^ in Nsp10; His^1275^ in Nsp11; Thr^1441^ in Nsp12; and Asn^34^ in GP5) were found in PRRSV2/CN/FJLX06-2021 compared to HuN4 and HuN4-F112. These results suggest that PRRSV2/CN/FJLX06-2021 has evolved from the HuN4-F112 strain under field conditions.

### 3.3. Pathogenicity Analysis of the PRRSV2/CN/FJLX06-2021

**Figure 2:**
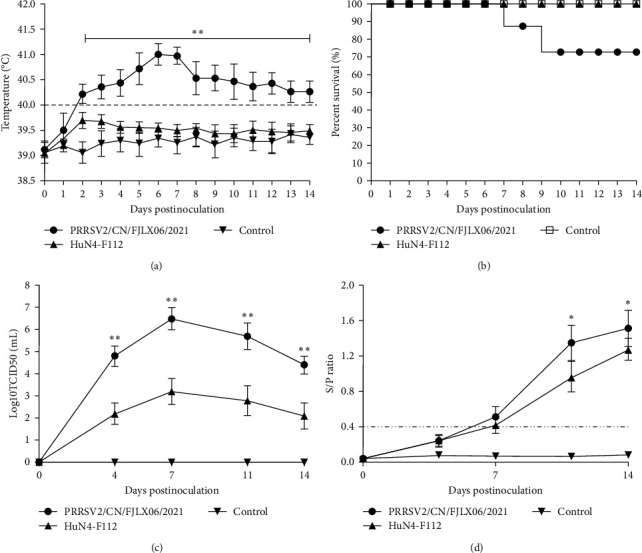
Rectal temperatures, survival curves, viremia, and PRRSV-special antibody levels of piglets in each group during the experiment. (a) Rectal temperatures of piglets in each group after challenge. The threshold of fever was set at 40.0°C. (b) Survival curves of the inoculated piglets in each group. (c) Virus titers in the sera of the inoculated piglets in each group. (d) PRRSV-specific N protein antibody levels in the sera of the inoculated piglets in each group. Data are the mean ± standard deviation (error bars). The asterisk indicates a significant difference between the PRRSV2/CN/FJLX06-2021-infected group and the HuN4-F112-inoculated group ( ^*∗*^*p* < 0.05;  ^*∗∗*^*p* < 0.01).

Piglets infected with PRRSV2/CN/FJLX06-2021 developed high fever from 2 dpi (average 40.2°C), with a peak body temperature of 41.1°C at 7 dpi, and the piglets remained hyperthermic throughout the experiment (Figure 2(a)). In addition to higher temperatures, the PRRSV2/CN/FJLX06-2021-infected group showed more severe clinical signs, such as dyspepsia, coughing, and shivering from 4 to 12 dpi. Two piglets in the PRRSV2/CN/FJLX06-2021-infected group died at 7 and 9 dpi, respectively, whereas all piglets in the HuN4-F112 and the negative control groups survived to the end of the experiment with normal rectal temperatures and behavior (Figure 2(b)). Piglets infected with PRRSV2/CN/FJLX06-2021 exhibited significantly higher average rectal temperatures than those of piglets inoculated with HuN4-F112 and the control group between 2 and 14 dpi (*p* < 0.01) (Figure 2(c)).

Serum samples were collected and assayed for viral load using IFA. As shown in Figure 2(c), the viral load of the PRRSV2/CN/FJLX06-2021-infected group continued to increase, reaching a peak of 10^6.5^ TCID50/mL at 7 dpi and then gradually decreased, but remained high at 14 dpi (10^4.5^ TCID50/mL), while the viral load of the HuN4-F112-inoculated piglets reached a peak of 10^3.2^ TCID50/mL at 7 dpi and then decreased. PRRSV was not detected in the negative control group for the entire duration of the study. The mean serum viral load of the PRRSV2/CN/FJLX06-2021 group was significantly higher than that of the HuN4-F112 group (*p* < 0.05). The specific antibodies of the PRRSV N protein in sera of piglets were measured by a commercial IDEXX ELISA kit. All piglets in PRRSV2/CN/FJLX06-2021-infected group seroconverted (S/P > 0.4) at 7 dpi. Two of the five piglets in the HuN4-F112-inoculated group seroconverted at 7 dpi, and the remaining three piglets seroconverted at 11 dpi. Moreover, the antibody level (mean S/P ratio values) of the PRRSV2/CN/FJLX06-2021-infected group was higher than that of the HuN4-F112-inoculated group, and statistically significant differences were observed at 11 and 14 dpi (*p* < 0.05). The PRRSV N protein antibody was negative in all of the piglets in the control group during the experiment. These above data indicated that PRRSV2/CN/FJLX06-2021 had higher replication efficiency in vivo than HuN4-F112 [29].

**Figure 3:**
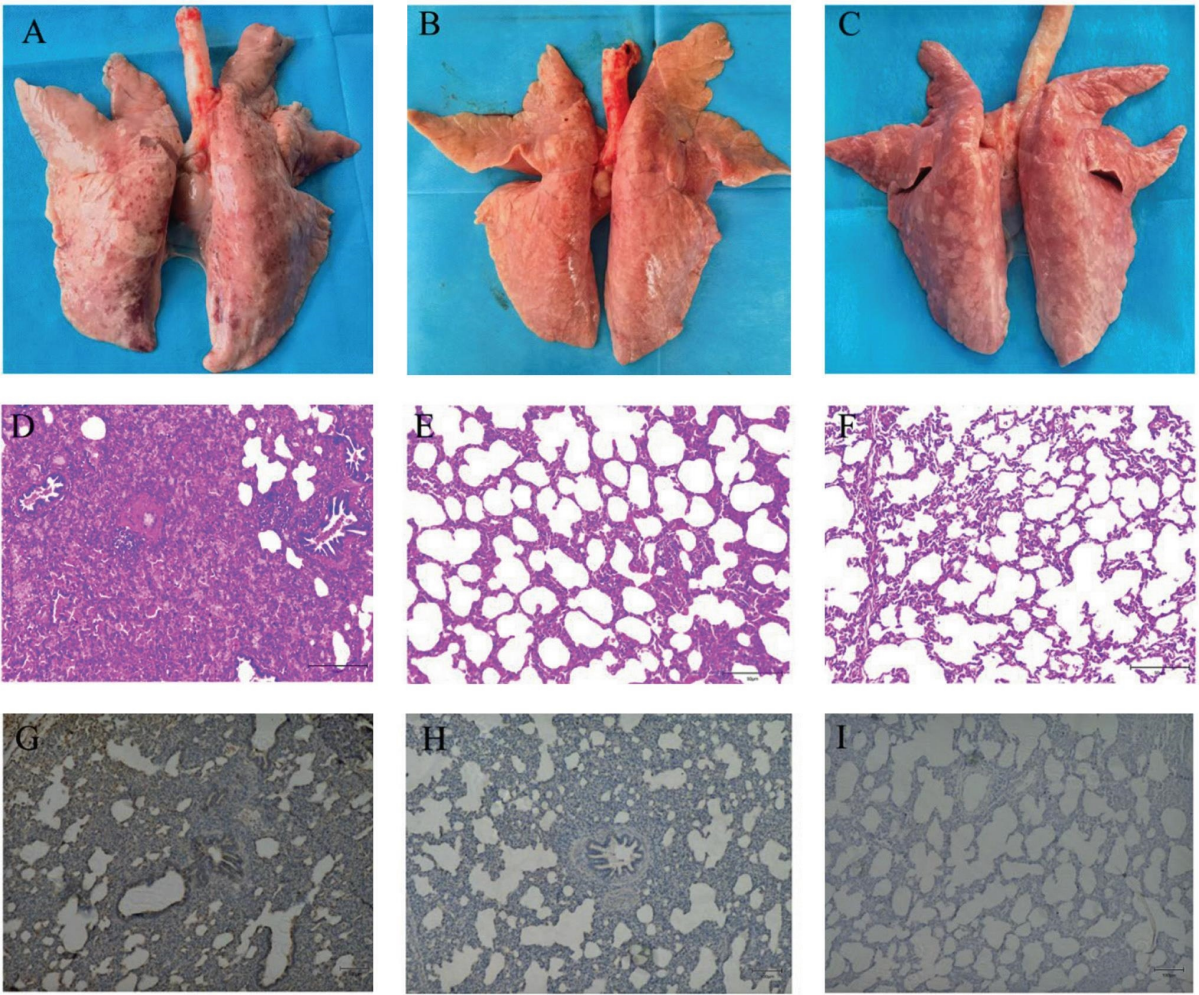
Gross lesions and microscopic lesions in the lung in each group. Gross pathological changes in the lung at 14 dpi in PRRSV2/CN/FJLX06-2021-infected group (A), HuN4-F112-inoculated group (B), and control group (C). Microscopic pathological changes in the lung at 14 dpi in PRRSV2/CN/FJLX06-2021-infected group (D), HuN4-F112-inoculated group (E), and control group (F). IHC examination for PRRSV antigen in the lungs of the PRRSV2/CN/FJLX06-2021-infected group (G), HuN4-F112-inoculated group (H), and control group (I).

**Figure 4:**
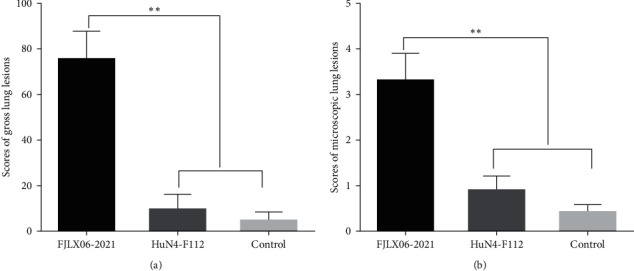
Scores of gross lung lesions (a) and microscopic lung lesions (b) of the challenged piglets. Asterisk indicates a significant difference between the PRRSV2/CN/FJLX06-2021-infected group and the HuN4-F112-inoculated and control groups ( ^*∗∗*^*p* < 0.01).

Gross lung lesions in piglets of the PRRSV2/CN/FJLX06-2021-infected group showed pulmonary consolidation and multifocality, together with scattered hemorrhagic spots (Figure 3(A)). No obvious lung gross lesions were observed in the HuN4-F112-inoculated group (Figure 3(B)). Meanwhile, microscopic lung lesions of piglets in the PRRSV2/CN/FJLX06-2021-infected group showed obvious interstitial pneumonia with severe pulmonary edema, characterized by marked alveolar septal thickening, alveoli disappearance, alveolar septa hemorrhage and fluid-filled alveolar spaces, and massive inflammatory cell infiltration within the lung tissues (Figure 3(D)). However, animals in the HuN4-F112-inoculated group showed only mild interstitial pneumonitis compared to the PRRSV2/CN/FJLX06-2021-infected group (Figure 3(E)). No gross or microscopic lung lesions were observed in the control group (Figure 3(C), and 3(F)). Meanwhile, the mean lung gross lesion and microscopic lesion scores of the piglets in the PRRSV2/CN/FJLX06-2021-infected group were significantly higher than those of the HuN4-F112-inoculated and the control groups (*p* < 0.01). On the contrary, the HuN4-F112-inoculated group had slightly higher levels of gross and microscopic lung lesions than the control group, but the differences were not significant (*p* > 0.05) (Figure 4). PRRSV antigens in lungs were examined by IHC. As shown in Figure 4, PRRSV-positive signal could be seen in the macrophages and alveolar wall cells in the lung tissue of PRRSV2/CN/FJLX06/2021-infected piglets, whereas less positive signals could be observed in the HuN4-F112-inoculated piglets. No PRRSV-positive signals were observed in control piglets.

## 4. Discussion

PRRS, particularly the outbreak of HP-PRRSV in 2006, has caused huge economic losses to the Chinese pig industry [16, 17]. Vaccination is one of the major strategies to control PRRS in China [8, 30, 31]. Since 2011, several MLV vaccines (HuN4-F112, JXA1-R, GDr180, and TJM-F92) derived from HP-PRRSV strains have been widely used in pig farms to control HP-PRRS [19, 31]. Unfortunately, under the pressure of vaccination, PRRSV has been mutating continuously and is becoming genetically diverse. In addition, MLV-derived PRRSV strains including HuN4-F112-like strains are frequently isolated in the field [19, 27, 31, 32, 33, 34]. Worryingly, MLV-like strains regained virulence during continuous field dissemination [2, 19, 34, 35, 36].

In the present study, a PRRSV strain PRRSV2/CN/FJLX06-2021 was isolated from field outbreaks of the disease that showed and had the highest nucleotide similarity to strain HeN1201 (HuN4-F112-like strain) [31]. Furthermore, there are 39 unique amino acid mutations within the HuN4-F112 genome. We discovered 29 of these amino acids in the corresponding locations of PRRSV2/CN/FJLX06-2021. In addition, there was no evidence of recombination in the PRRSV2/CN/FJLX06-2021 isolate by using RDP 4.10 software. A recent study showed that a highly attenuated strain JXwn06-P80 (derived from HP-PRRSV JXwn06) regained lethal virulence through transnasal inoculation in pigs or sequential transmission of primary PAM [2]. In addition, a retrospective survey found that this farm created a PRRS-positive stable herd by administering a low dose of the HuN4-F112 vaccine. Combined with the recent report described by Wang et al. [2], we concluded that PRRSV2/CN/FJLX06-2021 might be a revertant strain that has evolved from the vaccine strain HuN4-F112. Unnecessary mass vaccination with PRRS MLV strains can result in vaccine-derived strains in the field, raising concerns about possible reversion of virulence [2, 31]. Previous studies have reported the highly virulent MLV-like PRRSVs derived from JXA1-R [20]. In addition, HuN4-F112-like strains and TJM-F92-like strains are circulating in China, but their virulence has not been evaluated [20, 31, 32]. In this study, our study showed TJM-F92-like strain PRRSV2/CN/FJLX06-2021 is highly virulent for piglets, and to our knowledge, our study is the first report that HuN4-F112-derived PRRSV regained the virulence in field. In view of HP-PRRSV vaccine, viruses can evolve and reverse in the vaccinated pig farm. Therefore, HP-PRRSV MLV vaccination should be restricted in field to avoid the generation of novel PRRSV strains [31].

Persistent high fever and high mortality (20%–100%) were important signs of HP-PRRSV infection [16, 37]. In the present study, persistent high fever and 40% mortality were only found in the PRRSV2/CN/FJLX06-2021-infected group, these results indicate that PRRSV2/CN/FJLX06-2021 is a highly pathogenic strain. ORF1a gene of HP-PRRSV plays an important role in viral replication and lung damage *in vivo* and *in vitro* based on the reverse genetic system of HuN4 and HuN4-F112 [37]. In addition, Nsp9 and Nsp10 contribute to the fatal virulence of HP-PRRSV for piglets [22]. Moreover, Nsp3-8 and ORF5 are identified as major virulence determinants of PRRSV by constructing chimeric viruses with a swapped fragment from the highly virulent strain FL12 [38]. Similarly, in this study, PRRSV2/CN/FJLX06-2021 had seven out of nine reversion mutations located in Nsp2, Nsp7, Nsp9, Nsp10, and ORF5 compared to HuN4-F112, which may enhance the virulence of the PRRSV2/CN/FJLX06-2021 isolate. Certainly, we need to further identify the key amino acids responsible for the virulence of PRRSV2/CN/FJLX06/2021 in piglets using a reverse genetics system.

In conclusion, our studies indicate that PRRSV2/CN/FJLX06-2021 is an MLV-like strain that has evolved from MLV HuN4-F112 and exhibits high pathogenicity to piglets.

## Figures and Tables

**Table 1:** Representative PRRSV strains used in this study.

No.	Name	GenBank accession no.	Origin	No.	Name	GenBank accession no.	Origin
1	PRRSV2/CN/FJLX06/2021	OQ357724	China	19	RespPRRS MLV	AF066183	USA
2	JXwn06	EF641008	China	20	QYYZ	JQ308798	China
3	TJ	EU860248	China	21	NADC30	JN654459	USA
4	JXA1	EF112445	China	22	JX143	EU708726	China
5	HuN4	EF635006	China	23	CHsx1401	KP861625	China
6	JXA1 P80	FJ548855	—	24	FJZ03	KP860909	China
7	MLV-like TJbd14-1	KP742986	China	25	MLV-like HeN1201	MF689000	China
8	HENNAN-XINX	KF611905	China	26	MLV-like HeN1502	MF766473	China
9	CH-1a	AY032626	China	27	NT1	KP179402	China
10	CH-1R	EU807840	China	28	HeNan-A2	KJ002452	China
11	HB-1(sh)/2002	AY150312	China	29	JL580	KR706343	China
12	HB-2(sh)/2002	AY262352	China	30	HUB1	EF075945	China
13	VR-2332	U87392	China	31	HENXX-9	KY290748	China
14	PA8	AF176348	Canada	32	HeNan-A9	KJ546412	China
15	BJ-4	AF331831	China	33	FJ1402	KX169191	China
16	FJFS	KP998476	China	34	HEB-2013	KJ591659	China
17	GD-KP	KU978619	China	35	GD1404	MF669720	China
18	GM2	JN662424	China	36	LV	M96262	Netherlands

**Table 2:** Comparison of the nucleotide and amino acid identities of PRRSV2/CN/FJLX06/2021 with those of PRRSV reference strains.

PRRSV2/CN/FJLX06/2021	NADC30	FJZ03	NADC34	FJFS	GM2	QYYZ	VR2332	BJ-4	JXA1	TJ	HuN4	HeN1201	TJbd14-1	JXA1 P80	LV
	Lineage 1			Lineage 3 (QYYZ-like)			Lineage 5.1		Lineage 8.7 (HP-PRRSV)			Lineage 8.7 (MLV-like strain)			
Pairwise % identity to PRRSV2/CN/FJLX06/2021 (nt/aa)															
Nucleotides															
Complete genome	84.5	83.9	84.2	87.8	87.5	87.7	89.5	89.4	99.2	99.2	99.5	99.7	98.8	99.0	60.3
5′ UTR	93.1	92.1	93.0	93.6	93.6	95.8	95.8	95.8	100	97.4	100	99.5	99.5	100	66.3
ORF1a	81.2	80.7	80.8	86.1	84.7	85.1	87.4	87.3	99.1	99.2	99.4	99.6	98.7	99.0	55.8
ORF1b	87.6	86.9	86.4	90.1	90.7	90.3	91.1	91.1	99.4	99.4	99.6	99.8	99.0	99.3	66.3
ORF2-7	86.4	85.9	86.6	87.9	89.0	89.1	91.4	91.3	99.0	99.1	99.2	99.6	98.9	98.8	65.2
3′ UTR	90.0	91.2	89.3	93.3	93.3	86.7	87.3	88.7	100	100	100	100	100	98.7	72.6
Amino acids															
NSP1*α*	97.0	94.0	97.0	94.6	96.4	96.4	95.8	95.8	100	100	100	100	100	100	66.9
NSP1*β*	77.0	74.7	78.3	91.2	82.9	82.9	85.7	84.8	98.2	98.6	98.6	99.5	97.7	97.7	42.3
NSP2	68.4	67.3	66.8	78.4	76.3	76.8	77.1	76.2	98.0	98.2	98.8	98.9	96.3	97.7	33.2
NSP3	90.8	91.0	90.8	88.1	88.1	88.1	94.4	94.4	99.1	99.3	99.6	100	98.9	98.9	57.8
NSP4	92.6	92.2	94.1	90.7	91.7	91.2	93.1	93.1	98.5	98.5	98.5	99.0	98.0	98.0	61.6
NSP5	94.1	92.4	90.0	88.8	89.4	89.4	92.9	93.5	99.4	99.4	99.4	99.4	98.2	99.4	71.8
NSP6	93.8	93.8	87.5	100	100	100	93.8	93.8	100	100	100	100	100	100	75.0
NSP7	84.6	83.8	86.5	92.7	90.3	94.6	89.6	88.4	99.6	98.8	96.6	99.2	99.6	99.2	46.9
NSP8	95.6	95.6	93.3	95.6	100	100	100	100	100	100	100	100	100	100	68.9
NSP9	96.7	9,538	96.6	96.0	96.7	96.6	97.2	96.9	98.9	99.2	98.8	99.5	98.9	99.1	74.9
NSP10	95.7	95.2	95.5	94.3	96.6	97.5	96.4	96.4	99.3	99.5	100	99.8	99.1	99.5	64.9
NSP11	97.3	95.5	96.4	94.6	95.5	94.6	94.2	95.5	100	99.6	100	100	99.1	99.1	77.1
NSP12	96.1	96.1	91.5	94.1	96.7	96.1	95.4	100	100	100	100	100	100	100	43.7
ORF2a/GP2	85.9	86.7	85.5	87.5	89.1	89.5	92.2	91.4	98.0	98.0	97.7	100	98.4	96.9	61.4
ORF2b/E	93.2	90.4	86.3	76.7	89.0	89.0	90.4	90.4	97.3	97.3	97.3	100	97.3	98.6	72.9
ORF3/GP3	79.5	78.0	81.9	83.1	87.0	86.2	85.0	85.8	97.6	97.6	98.0	98.4	97.6	97.2	56.1
ORF4/GP4	89.9	89.3	90.4	89.9	92.7	93.3	91.0	92.1	95.5	97.2	97.8	100	97.8	97.2	70.8
ORF5/GP5	85.5	86.5	86.5	82.5	82.0	82.5	88.0	86.0	99.0	99.0	99.0	98.0	98.0	97.5	57.1
ORF5a	82.6	82.6	84.8	78.3	76.1	76.1	82.6	82.6	100	100	100	100	100	100	45.5
ORF6/M	93.1	92.5	93.7	96.6	96.0	96.6	97.7	97.7	99.4	99.4	99.4	100	99.4	98.9	79.8
ORF7/N	90.2	89.4	90.2	91.1	88.6	91.1	94.3	94.3	99.2	99.2	99.2	98.4	98.4	99.2	64.2

**Table 3:** The amino acid mutations within the genomes of the PRRSV2/CN/FJLX06-2021 together with HuN4, HuN4-F112, HeN1201, and HeN1502.

ORF	Amino acids	aa position	HuN4	HuN4-F112	PRRSV2/CN/FJLX06-2021	HeN1201^a^	HeN1502^a^
ORF1a	Nsp1*α*	200	S	**F**	**F**	F	S
		343	V	**A**	**A**	A	V
	Nsp2	401	H	**R**	**R**	R	R
		477	T	**I**	**T**	I	I
		799	D	**N**	**N**	N	N
		894	V	**A**	**A**	A	A
		1009	S	**P**	**S**	P	P
		1090	N	**S**	**S**	S	S
		1166	C	**S**	**S**	S	S
		1481	T	**A**	**A**	A	A
	Nsp3	1674	A	**V**	**V**	V	V
	Nsp4	2008	A	**V**	**V**	V	V
	Nsp7	2250	I	V	I	V	I

ORF1b	Nsp9	22	T	A	T	T	T
		58	Y	**H**	**H**	H	H
		123	A	**V**	**V**	V	V
		217	S	**P**	**P**	S	S
		490	R	**Q**	**Q**	Q	Q
		505	I	**L**	**L**	L	I
		630	V	**F**	**F**	F	F
	Nsp10	702	K	R	K	K	K
		712	E	G	E	E	E
	Nsp11	1275	H	Y	H	Y	Y
	Nsp12	1441	T	I	T	T	T

ORF2a	GP2	50	Y	**S**	**S**	S	S
		118	I	**V**	**V**	V	V
		120	E	**D**	**D**	D	D
		177	S	**A**	**A**	A	A
		250	T	**I**	**I**	I	I

ORF2b	E	9	D	**N**	**N**	N	N
		48	M	**V**	F	V	V

ORF3	GP3	48	M	**V**	**V**	V	V

ORF4	GP4	43	D	N	G	G	N
		67	S	**P**	**P**	P	P
		124	I	**V**	**V**	V	V
		129	V	**I**	**I**	I	I

ORF5	GP5	34	N	D	N	D	D
		196	Q	**R**	**R**	R	R

ORF6	M	63	V	**A**	**A**	A	A

The identical mutation sites between PRRSV2/CN/FJLX06-2021and HuN4-F112 are marked with bold. ^a^MLV-like strain evolved from the vaccine virus HuN4-F112.

## Data Availability

The data used to support the findings of this study are included within the article and at GenBank database.

## Supplementary Materials

Supplementary Materials Figure S1: IFA identification of PRRSV by MARC-145 cells. Figure S2: recombination analysis of PRRSV2/CN/FJLX06/2021 genome using RDP4.10 software.
