# Supplementary material for: Characterization and Pathogenicity of a Porcine Reproductive and Respiratory Syndrome Virus Strain with Strong Homology to a HP-PRRSV Vaccine Strain in the Field
Source: Transbound Emerg Dis. 2024 Jun 21;2024:1297975. doi: 10.1155/2024/1297975 (PMC12017044; doi:10.1155/2024/1297975)
Supplement: Supplementary Materials — Figure S1: IFA identification of PRRSV by MARC-145 cells. Figure S2: recombination analysis of PRRSV2/CN/FJLX06/2021 genome using RDP4.10 software. [file 1297975.f1.pdf]

Figure S1

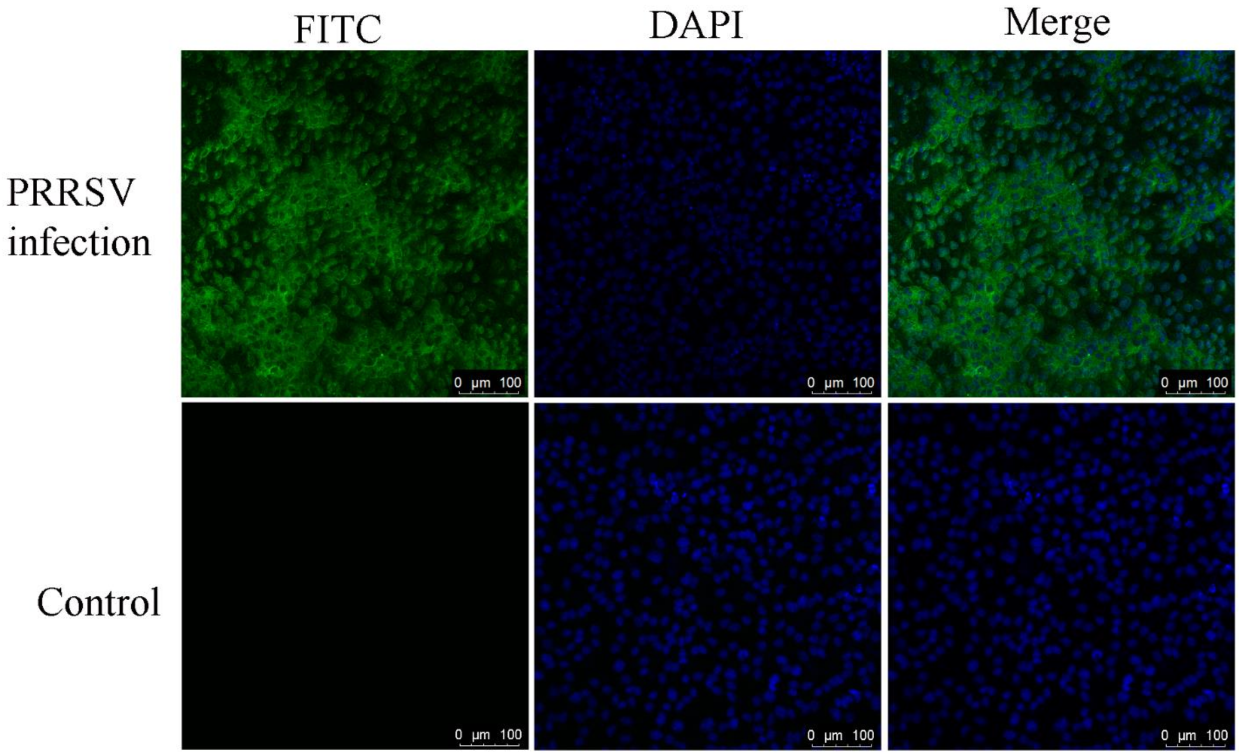

Fig S1 IFA identification of PRRSV by MARC-145 cells

Figure S2

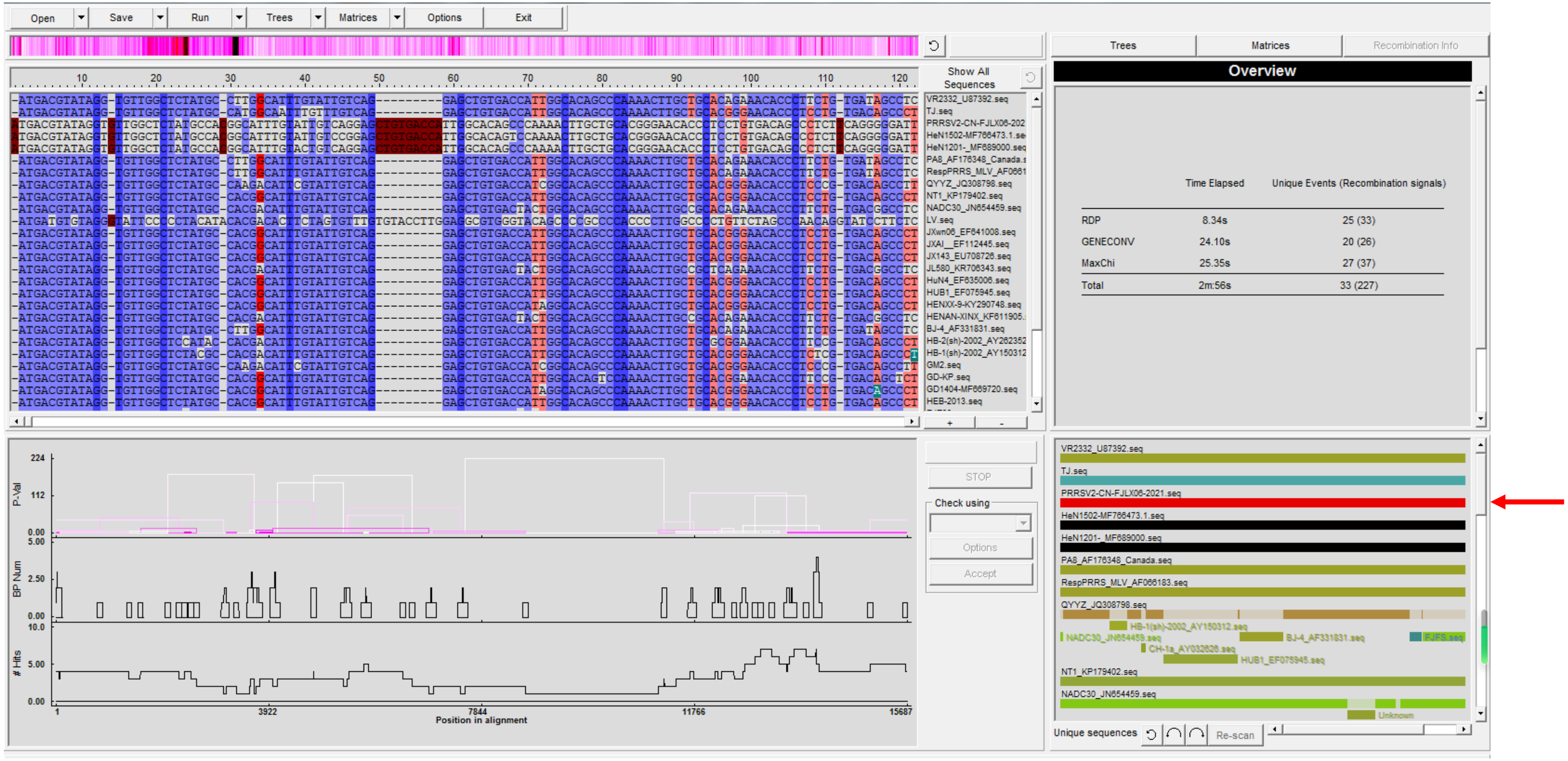

Fig S2 Recombination analysis of PRRSV2/CN/FJLX06/2021 genome using RDP4.10 software.
